# Single-cell atlas reveals a pro-metastatic RELB+ neutrophil-myeloid subset underlying lymph node metastasis in EGFR-wildtype LUAD

**DOI:** 10.3389/fcell.2026.1766211

**Published:** 2026-02-17

**Authors:** Fasheng Li, Xiao Yang, Ang Li, Yutao Pang, Hongfei Zhang, Liyao Lin, Dong Wu

**Affiliations:** Division of Thoracic Surgery, Affiliated Hospital of Guangdong Medical University, Zhanjiang, China

**Keywords:** EGFR-wildtype, lung adenocarcinoma, lymph node metastasis, RelB, tumormicroenvironment

## Abstract

**Background:**

Lymph node metastasis is a critical event in the progression of EGFR wildtype lung adenocarcinoma (LUAD), a subtype lacking effective targeted therapies and associated with poor prognosis. The specific myeloid cell subsets and molecular mechanisms driving LNM in this context remain poorly understood.

**Methods:**

To elucidate the cellular drivers of LNM, we performed an integrated analysis of multi-cohort single-cell RNA sequencing data from EGFR-wildtype LUAD patients and bulk transcriptomic data from The Cancer Genome Atlas (TCGA). Pathological lymph node status from the bulk RNA-seq cohort was mapped to the single-cell transcriptomes using the UCell algorithm. Myeloid cell heterogeneity was analyzed via sub-clustering, and the transcription factor regulon activity was inferred using pySCENIC. The functional role of the key regulator RELB was validated using siRNA-mediated knockdown in neutrophil-tumor cell co-culture systems, assessed by qPCR, proliferation, clonogenic, migration, and invasion assays. A RELB signature score was constructed based on its target genes and validated in independent cohorts for prognostic, immunotherapeutic, and drug sensitivity assessment.

**Results:**

Myeloid cells were significantly enriched in the LNM group. The process of sub-clustering identified a pro-metastatic subset of ELANE-positive neutrophils that was specifically expanded in LNM samples, linked to advanced N stage, higher clinical stage, and shorter overall survival. RELB was identified as a central regulator of ELANE + Neu through transcription factor analysis, showing significantly heightened regulon activity in LNM. Using a neutrophil-tumor cell co-culture system, we found that RELB knockdown in neutrophils attenuated inflammatory signaling in tumor cells and subsequently reduced their proliferative, clonogenic, migratory, and invasive capacities. A high score of the RELB signature was a strong predictor of poor prognosis and was associated with pro-tumorigenic pathways, and the creation of an immunosuppressive microenvironment. Additionally, RELB scores were associated with lower tumor mutational burden, poorer response to immunotherapy, different drug sensitivity patterns, and increased expression of several antibody-drug conjugate targets.

**Conclusion:**

The study highlights a pro-metastatic ELANE + neutrophil subpopulation, with RELB acting as its primary transcriptional regulator. The RELB signature may serve as a biomarker for prognosis and treatment response prediction, indicating a potential target for precision treatment in EGFR wildtype LUAD.

## 1 Introduction

Lung adenocarcinoma (LUAD) is the most common histological form of non-small cell lung cancer (NSCLC). Approximately 40% of lung cancer cases diagnosed globally each year are of this type, which has a 5-year survival rate of less than 25% (Bray et al., 2024; Deschênes, 2025). Lymph node metastasis (LNM) significantly raises the risk of death, even for patients diagnosed early (Chen X. et al., 2025; Xia et al., 2025). Therefore, understanding the cellular and molecular processes behind LNM is crucial for improving clinical outcomes.

Within the realm of precision oncology, determining EGFR mutation status is essential for crafting treatment strategies for LUAD (Borgeaud et al., 2025). Patients with EGFR sensitizing mutations, such as exon 19 deletions or the exon 21 L858R mutation, show a significant survival benefit when administered EGFR tyrosine kinase inhibitors (TKIs) (Ramalingam et al., 2020; Lu et al., 2022). By contrast, EGFR wildtype lung adenocarcinoma represents 30%–55% of cases and remains a major therapeutic challenge. The median overall survival (OS) in this patient group is only 12–18 months, roughly half that of patients with EGFR mutations. Moreover, these patients lack effective targeted therapies and exhibit a higher rate of LNM (Deschênes, 2025; Borgeaud et al., 2025; Wu et al., 2025). The current emphasis in basic and translational research is largely on EGFR-mutant LUAD, particularly with respect to TKI resistance mechanisms and the tumor microenvironment alterations during targeted therapy (Zhang X. et al., 2025; Dai et al., 2024). However, a detailed study of the specific mechanisms behind lymph node metastasis in EGFR wildtype lung adenocarcinoma has not been fully carried out.

The presence of myeloid cells in the tumor microenvironment is vital for the development of cancer, This varied group includes various functional types like tumor-associated macrophages (TAMs), dendritic cells (DCs), neutrophils, and myeloid-derived suppressor cells (MDSCs) (Barry et al., 2023; Xiao and Yu, 2021). While these subsets can trigger anti-tumour immunity through antigen presentation, they frequently facilitate metastasis by suppressing T-cell function, promoting angiogenesis and altering the extracellular matrix (Zhang C. et al., 2025; Kusmartsev, 2025). Although increased myeloid cell infiltration in LUAD is associated with LNM and poor outcomes (Bai et al., 2025; Xing et al., 2025; Ouyang et al., 2025), the drivers of LNM in EGFR wildtype LUAD remain unclear. Specifically, the responsible myeloid subsets, the transcriptional networks governing their pro-metastatic functions, and their roles in fostering an immunosuppressive microenvironment are not well defined.

Breakthroughs in single-cell RNA sequencing (scRNA-seq) technology provide a high-resolution tool for dissecting TME heterogeneity and have identified numerous functionally distinct rare cell subsets in the LUAD immune landscape (Ren et al., 2024; Bischoff et al., 2021). While scRNA-seq has identified the dynamic balance between CXCL9+ and TREM2+ TAMs as a key factor in LUAD invasion and metastasis (Ren et al., 2024), the study did not specifically analyze this balance in relation to EGFR status. A separate study combined single-cell RNA sequencing (scRNA-seq) with machine learning techniques to elucidate the significant role of an iron metabolism risk score in epithelial cells throughout the progression of LUAD (Zhao et al., 2025). This research also facilitated the development of a predictive model for chemotherapy resistance (Zhao et al., 2025). However, this work neither accounted for differences across EGFR subtypes nor explored the regulatory role of immune cells in the LNM of EGFR-wildtype LUAD. These identified gaps underscore the urgent need for focused research into EGFR-wildtype LUAD to elucidate the underlying mechanisms driving its lymph node metastasis.

To address these knowledge gaps, we integrated seven scRNA-seq datasets from EGFR-wildtype LUAD patients with TCGA bulk transcriptomic data to systematically analyze the cellular and molecular drivers of LNM. We mapped the LNM status from the TCGA cohort to the single-cell level using the UCell algorithm and delineated the TME composition of EGFR wildtype LUAD. Proportion and deconvolution analyses confirmed significant enrichment and higher infiltration scores of myeloid cells in LNM group. Additional sub-clustering revealed that ELANE-positive neutrophils (ELANE + Neu) is a crucial pro-metastatic myeloid group. The pySCENIC analysis of transcription factor regulons pinpointed RELB as the main transcription factor controlling the pro-metastatic role of ELANE + neutrophils. There was a significant enrichment of RELB target genes in NF-κB signaling and inflammatory response pathways. In addition, functional assays indicated that the suppression of RELB in neutrophils diminished inflammatory signaling in tumor cells and reduced their proliferative, clonogenic, migratory, and invasion abilities. A signature score originating from RELB target genes was eventually developed and verified across different independent cohorts. The score demonstrated prognostic importance and the capability to forecast immunotherapeutic responses and drug sensitivity.This investigation progresses the knowledge of metastatic biology in EGFR wildtype LUAD, presenting potential biomarkers and innovative treatment methods for patients with restricted treatment alternatives.

## 2 Methods

### 2.1 Data collection

We integrated publicly available single-cell and bulk RNA sequencing datasets. A total of seven single-cell RNA-seq (scRNA-seq) datasets were obtained from the Gene Expression, including: E-MTAB-13526, GSE164789, GSE207422 (Hu et al., 2023), GSE117570 (Wang et al., 2021), GSE241934 (Chen M. et al., 2025), GSE131907 (Kim et al., 2020) and GSE148071 (Wu et al., 2021). All datasets were downloaded from the GEO repository and preprocessed using standardized workflows. Three bulk RNA-seq datasets were incorporated for integrative analysis. The TCGA cohort (n = 133), with gene expression profiles and clinical annotations obtained from the National Cancer Institute’s Genomic Data Commons (GDC) Data Portal. Data regarding clinical outcomes and survival probabilities for the GSE115002 (Liao et al., 2025) and GSE13213 (Tomida et al., 2009) cohorts were retrieved from the GEO database to ensure the consistency and comparability of clinical variables. Data on immune checkpoint inhibitors in lung cancer were obtained from su2c cohort (Ravi et al., 2023). All dataset information were collected in Supplementary Table S1.

For RNA-seq datasets, gene expression values were transformed to log2 (TPM + 1), whereas for microarray datasets, normalized expression matrices provided by the original studies were used. The batch effects across datasets were eliminated by means of the implementation of the Combat algorithm from the SVA R package (Zhang et al., 2020). Following correction, the three bulk cohorts were merged into a unified meta-cohort for downstream integrative analyses and feature extraction.

### 2.2 Data processing and analysis

For single-cell transcriptomic analysis, initial quality control was performed in accordance with the following criteria: (1) removal of ribosomal and kinase genes; (2) mitochondrial gene content <15%. The detail information of scRNA-seq data were collected in Supplementary Table S2. The raw expression matrix was subsequently normalized to eliminate technical bias. To address batch effects arising from multi-sample integration, Harmony algorithm was applied to the principal component analysis (PCA) results for batch correction. The top 30 principal components were selected for downstream analysis. Unsupervised clustering was conducted to identify cell populations, and UMAP was used for dimensionality reduction and visualization. To ensure the accuracy of cell-type annotation, clusters were annotated at a resolution of 0.6 using lineage-specific marker genes. The annotation of each cluster to specific cell types was based on established canonical markers. Myeloid subpopulations were then subjected to further annotation, after which differential gene expression analysis was performed using the COSG algorithm. Pathway enrichment analysis was conducted for each subpopulation using the KEGG database via the clusterProfiler R package (v4.12.1), with Benjamini–Hochberg false discovery rate correction.

Samples from the TCGA cohort were stratified based on pathological lymph node status (PN), with N0M0 samples designated as the control group and N > 0M0 samples designated as the treatment group. The differential expression analysis between the treatment and control groups was performed using the limma package (v3.56.2). The ranking of genes was based on adjusted p-values and log_2_ fold changes. The top 100 up- and downregulated genes were selected to construct up- and downregulated gene sets, respectively. Subsequently, gene set enrichment scores were calculated in single-cell RNA sequencing data using the irGSEA framework, based on these two gene sets. Based on the up- and downregulated gene sets, cells with enrichment scores in the top 10% were defined as LNM cells and non-LNM cells, respectively.

### 2.3 Bulk deconvolution analysis

To evaluate the functional contributions of distinct cell types within bulk RNA-seq datasets, deconvolution analysis was performed on nine annotated myeloid subpopulations using InstaPrism (version 0.1.6) to assess their infiltration levels. Based on the estimated infiltration abundance of each cell type, samples were stratified for prognostic evaluation, and survival analyses were conducted within the bulk cohorts. Specifically, infiltration scores were compared between different nodal statuses (N > 0 vs. N = 0) and clinical stages (Stage I-II vs. Stage III) using the Wilcoxon rank-sum test.

### 2.4 Transcription factor regulator analysis

Regulatory network inference and transcription factor (TF) activity analysis were conducted using the pySCENIC framework (v0.11.2) (Van de Sande et al., 2020) with default parameters. Initially, GRNBoost2 was applied to construct gene regulatory networks by identifying co-expression modules and putative TF-target gene relationships. RcisTarget was then used to perform motif enrichment analysis to confirm TF binding site enrichment. Regulon activity scores were subsequently computed using the AUCell algorithm. For downstream analysis, only the top ten most specific regulons were retained for each cell subtype. To identify RELB target genes, the top 50 genes ranked by importance score were selected. The irGSEA was performed at the single-cell level to evaluate enrichment patterns across individual cells, with these RELB target genes being the basis for the study.

### 2.5 Differential expression analysis

The limma package (Ritchie et al., 2015) was used to identify genes differentially expressed between the high- and low-RELB score groups. KEGG pathway enrichment analysis was conducted via GSEA using the gseKEGG function in clusterProfiler (v4.12.1) (Demirel et al., 2020), with multiple testing correction performed using the Benjamini–Hochberg false discovery rate (FDR) method.

### 2.6 Cell culture and transfection

Human neutrophil cells (Cat# JN-CC6058) was obtained from Jining Bio Co., LTD. The human non-small cell lung cancer (NSCLC) cell lines A549 (Cat# CBP60053) and H1299 (Cat# CBP60053) were also purchased from Cobioer Biosciences Co., LTD. Upon thawing, all cell lines were centrifuged at 1,000 rpm for 5 min to remove the upper layer, followed by resuspension in complete DMEM medium supplemented with 10% fetal bovine serum (FBS) and 1% penicillin–streptomycin. Cells were maintained in a humidified incubator at 37 °C with 5% CO_2_. The culture medium was replaced every 2 days, and subculturing was performed every 3 days.

For gene silencing experiments, neutrophils cells were transfected with two independent small interfering RNAs targeting RELB (siRELB#1 and siRELB#2), as well as a negative control siRNA (siCtrl), using Lipofectamine™ RNAiMAX transfection reagent according to the manufacturer’s instructions. Cells were harvested 24 h post-transfection for downstream analyses. The siRNA sequence, used were as follows: siCtrl (UUC​UCC​GAA​CGU​GUC​ACG​U), siRELB#1 (GAAGAAGGAAAUUGAAGCU), siRELB#2 (GCU​ACG​GUG​UGG​ACA​AGA​A).

### 2.7 Reverse transcription and qPCR

Total RNA was extracted from cultured cells using TRIzol reagent according to the manufacturer’s protocol. RNA concentration and purity were assessed spectrophotometrically. Reverse transcription was performed using the ReverTra Ace qPCR RT Kit, following standard procedures. Quantitative real-time PCR (qPCR) was conducted using the 7500 Fast DX Real-Time PCR System with gene-specific primers. The primer sequences were in Supplementary Table S3. Gene expression levels were quantified using the 2^−ΔΔCt method, with β-actin serving as the internal control. All qPCR reactions were performed in triplicate, and expression differences were statistically analyzed.

### 2.8 Cell Counting Kit-8 (CCK-8) assay

Human neutrophil cells were seeded into 96-well plates. Cell viability was assessed at 0, 24, 48, and 72 h post-transfection using the Cell Counting Kit-8 (CCK-8). At each time point, 10 μL of CCK-8 solution was added to each well, followed by incubation at 37 °C for 2 h. Absorbance was measured at 450 nm using a microplate reader to evaluate cell viability. All measurements were performed in triplicate.

### 2.9 Clonogenic assay

Human neutrophil cells transfected with siRELB#1 or siRELB#2 were co-cultured with A549 or H1299 cells for 14 days, or until most colonies contained more than 50 cells. During the incubation period, the culture medium was replaced every 3 days. At the end of the experiment, cells were washed once with phosphate-buffered saline (PBS), fixed with 4% paraformaldehyde, and stained with crystal violet. Colony images were captured using a digital camera, and colony formation was quantified for analysis.

### 2.10 Transwell assays

A549 and H1299 cells were enzymatically dissociated, rinsed, and resuspended in serum-free medium at 4 × 10^6^ cells/mL. Subsequently, 500 μL of human neutrophils were placed in the lower chambers of 24-well plates, upon which Matrigel-coated Transwell inserts (8.0-μm pores) were mounted to facilitate direct contact between the 2 cell populations. Subsequently, 200 μL of A549 or H1299 cell suspension (8 × 10^5^ cells) was added to the upper chamber. After 24 h of incubation, non-migrated and non-invaded cells on the upper surface of the membrane were carefully removed. Cells on the lower surface were fixed with methanol, stained with crystal violet, and quantified under a light microscope at 100× magnification. Three representative fields were selected per insert for statistical analysis. Graphs were generated and statistical significance was determined using GraphPad Prism.

### 2.11 Statistical analysis

All statistical analyses were performed using R software (v 4.3.1). Survival analyses were conducted using the survival (v3.5–7) and survminer (v0.4.9) packages. Kaplan–Meier survival curves were generated to compare survival outcomes, and Cox proportional hazards regression models were used to estimate hazard ratios. Statistical significance was assessed using the Wilcoxon rank-sum test, with multiple comparisons adjusted via the Holm method. Differences in qPCR expression levels and cell counts were evaluated using unpaired t-tests. The infiltration scores difference between different nodal statuses (N > 0 vs. N = 0) and clinical stages (Stage I-II vs. Stage III) were accessed using the Wilcoxon rank-sum test. Drug-target pathway enrichment was assessed using hypergeometric testing, with p-values corrected for multiple comparisons using the Benjamini–Hochberg (BH) method.

## 3 Results

### 3.1 Single-cell atlas of EGFR-wildtype LUAD identifies myeloid cell enrichment in lymph node metastasis

**FIGURE 1:**
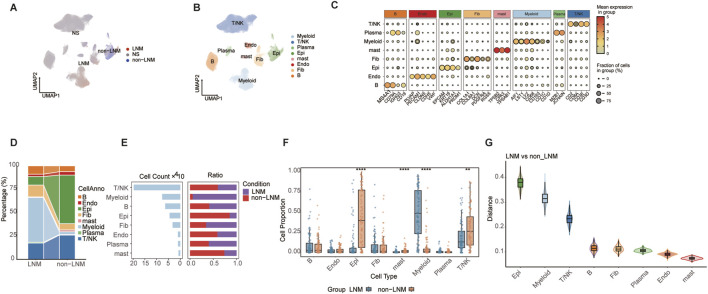
Single-cell atlas of LUAD identifies myeloid cell enrichment in lymph node metastasis. **(A)** UMAP plot for defining LNM, non-LNM, and NS cells of integrated single-cell data. **(B)** UMAP projection of integrated single-cell data, colored by major cell types. **(C)** Dot plot showing expression of canonical marker genes for each cell type. **(D)** Sankey diagram illustrating proportional shifts of cell types between LNM and non-LNM groups. **(E)** Bar plot quantifying cell numbers and proportions of each cell type in LNM and non-LNM groups. **(F)** Box plots comparing infiltration scores of each cell type between LNM and non-LNM groups across TCGA samples. **(G)** Box plots showing distribution distances between LNM and non-LNM cells within each cell type on UMAP space. Data are presented as mean ± SD. **P < 0.01, ****P < 0.0001 by wilcoxon rank-sum test **(F)**.

To decipher the cellular basis of lymph node metastasis (LNM) in EGFR-wildtype lung adenocarcinoma (LUAD), we integrated multiple single-cell RNA sequencing datasets from this patient subset. The batch correction was performed using Harmony algorithm (Supplementary Figure S1A). We employed the UCell algorithm to project the LNM status, defined from bulk RNA-seq data of the TCGA-LUAD (EGFR-wildtype) cohort, onto the integrated single-cell transcriptomes. This approach successfully defined LNM, non-LNM, and NS cells (Figure 1A). The UCell scores for LNM-upregulated and LNM-downregulated gene sets were visualized on UMAP plots for each cell (Supplementary Figure S1B,D) and the distribution of these scores across cell types were displayed using violin plots (Supplementary Figure S1C,E). Notably, the myeloid subpopulation exhibited the highest enrichment for the LNM-upregulated gene set (Supplementary Figure S1C). UMAP visualization revealed the comprehensive cellular composition of the EGFR-wildtype tumor microenvironment (Figure 1B), validated by the expression of canonical cell-type marker genes (Figure 1C). Proportional analysis identified myeloid cells as significantly enriched in the LNM group, a shift illustrated by sankey and bar plots (Figures 1D,E). This indicates a specific association between myeloid cell abundance and the LNM status in this subtype. Deconvolution analysis of bulk TCGA data further confirmed a significantly higher infiltration score for myeloid cells in the LNM group (Figure 1F).

Additionally, the analysis of distribution distance on the UMAP space suggested that myeloid cells stayed in a relatively stable state between LNM and non-LNM conditions (Figure 1G). This suggests that the observed enrichment is not due to major transcriptional state shifts but rather an expansion of a conserved myeloid population. Collectively, these outcomes designate myeloid cells as a significant cell population related to LNM in EGFR-wildtype LUAD, encouraging their examination in upcoming analyses.

### 3.2 Myeloid heterogeneity analysis reveals ELANE + Neu as a key pro-metastatic subpopulation in EGFR-wildtype LUAD

**FIGURE 2:**
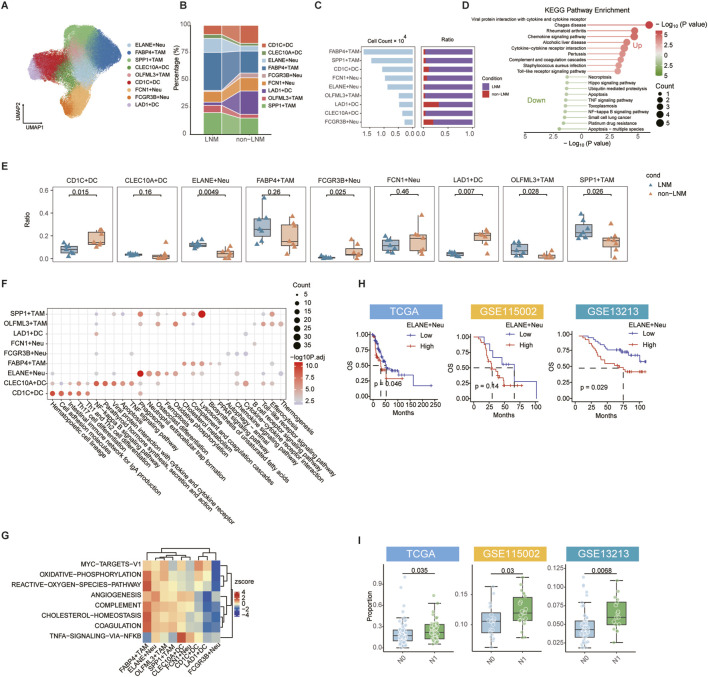
Myeloid heterogeneity analysis identifies ELANE + Neu as a key pro-metastatic subpopulation. **(A)** UMAP plot of myeloid cells re-clustered into 9 distinct subpopulations. **(B)** Sankey diagram depicting proportional changes of myeloid subpopulations between LNM and non-LNM groups. **(C)** Bar plot displaying cell numbers and proportions of each myeloid subpopulation. **(D)** KEGG enrichment analysis of DEGs in myeloid cells between LNM and non-LNM groups. **(E)** Box plots comparing proportions of each myeloid subpopulation across single-cell samples. **(F,G)** Functional enrichment analyses of DEGs specific to each myeloid subpopulation: KEGG pathways **(F)** and Hallmark gene sets **(G)**. **(H)** Kaplan-Meier analysis of overall survival based on optimal ELANE + Neu infiltration score in TCGA, GSE115002, and GSE13213 cohorts. **(I)** Comparison of ELANE + Neu infiltration scores across different N stages. Data are presented as mean ± SD. ns, not significant; ^*^ *P* < 0.05, ^**^ *P* < 0.01, ^***^ *P* < 0.001 by two-tailed wilcoxon test **(E,I)**. P values were determined by log-rank test **(H)**.

By performing sub-clustering, we aimed to analyze the heterogeneity of myeloid cells in the EGFR-wildtype context and identify specific subsets that drive LNM, leading to the identification of nine different myeloid subpopulations (Figure 2A). Proportional analysis indicated a distinct increase in the ELANE + Neu subpopulation within the LNM group, whereas other subsets like FCGR3B + Neu and LAD1+DC exhibited relative decreases (Figures 2B,C). The data suggests a targeted growth of ELANE + Neu within metastatic environments. Pseudo-bulk analysis of differentially expressed genes in LNM myeloid cells underscored their participation in inflammatory and immunosuppressive pathways (Figure 2D). This functional bias indicates a role in establishing a supportive microenvironment for metastasis. The analysis of individual samples showed that ELANE + Neu was significantly higher in LNM samples, with notable alterations in other subpopulations, including an increase in SPP1+TAM and OLFML3+TAM and a decrease in CD1C + DC, FCGR3B_Neu, and LAD1+DC (Figure 2E). This confirms the sample-level consistency of the ELANE + Neu expansion. Pathway analyses, including KEGG and Hallmark, provided additional insights into the specific functional roles of each myeloid subpopulation (Figures 2F,G). Specifically, ELANE + Neu was notably enriched in oxidative phosphorylation, phagocytosis, and ferroptosis, indicating a metabolic and inflammatory profile that supports metastasis. This unique functional profile potentially underlies its pro-metastatic capacity. To evaluate the clinical relevance of these subpopulations, we deconvoluted their infiltration scores into multiple independent EGFR-wildtype cohorts and performed survival analysis. A high ELANE + Neu infiltration score was significantly associated with shorter overall survival (Figure 2H). This establishes a direct link between ELANE + Neu abundance and poor patient outcome. Clinicopathological correlation further confirmed that this score was elevated in patients with advanced N stage and higher clinical stage (Figure 2I; Supplementary Figure S2).

Together, these results identify ELANE + Neu not merely as an associated cell type but as a pivotal myeloid subpopulation driving metastasis and poor prognosis in EGFR-wildtype LUAD. They also highlight the unique functional and metabolic profile of this subset within the tumor microenvironment.

### 3.3 The transcription factor RELB promotes a pro-metastatic program in ELANE + Neu cells

**FIGURE 3:**
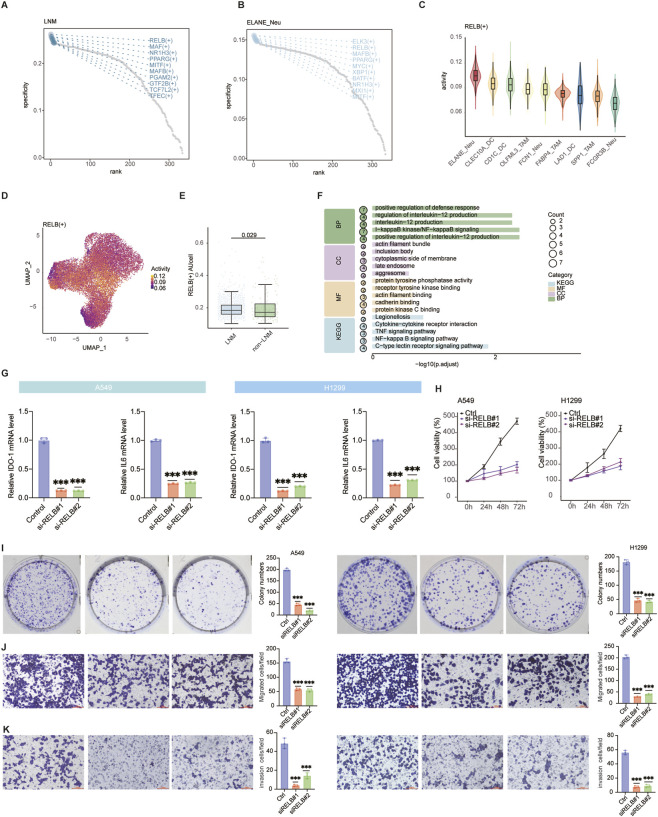
The transcription factor RELB drives a pro-metastatic program in ELANE + Neu. **(A)** Top transcription factors specifically enriched in myeloid cells from LNM group, ranked by regulon specificity score. **(B)** Top TFs specifically enriched in ELANE + Neu subpopulation, ranked by regulon specificity score. **(C)** Violin plot comparing activity of RELB (+) regulon between ELANE + Neu and other myeloid subpopulations. **(D)** Feature plot showing spatial activity of RELB (+) regulon across all myeloid cells on UMAP. **(E)** Box plot comparing RELB (+) regulon activity score (AUCell) between LNM and non-LNM groups. **(F)** KEGG and GO pathway enrichment analysis of genes targeted by RELB. **(G)** Expression levels of NF-κB-related genes (IDO-1 and IL-6) in A549 and H1299 tumor cells after coculture with RELB-knockdown neutrophils. **(H–K)** Functional assays in A549 and H1299 cells following coculture with RELB-knockdown neutrophils: **(H)** Cell proliferation, **(I)** Colony formation, **(J)** Migration, and **(K)** Invasion. Data are presented as mean ± SD. Statistical significance was determined by two-tailed Wilcoxon test **(C,E)** or two-tailed t-test **(G–K)**. ^*^ *P* < 0.05, ^***^ *P* < 0.001.

To elucidate the intrinsic regulatory mechanism governing the pro-metastatic function of the ELANE + Neu subpopulation, we performed transcription factor (TF) regulon analysis using pySCENIC. The results identified RELB as a top TF specifically enriched not only in the LNM group (Figure 3A) but also within the ELANE + Neu subpopulation itself (Figure 3B). This dual enrichment strongly suggests RELB is a key regulator specific to this pro-metastatic context and cell type. The activity of the RELB (+) regulon was significantly elevated in ELANE + Neu cells (Figure 3C) and across the LNM myeloid compartment (Figure 3D), with its activity score significantly higher in the LNM group (Figure 3E). This confirms heightened RELB-driven transcriptional activity in metastasis-associated myeloid cells. Consistent with its established role in promoting metastasis and immunosuppression in other cancer types such as hepatocellular carcinoma and diffuse large B-cell lymphoma (Tan et al., 2015; Nuan-Aliman et al., 2022), our findings highlight RELB as a key transcriptional regulator in EGFR-wildtype LUAD. Enrichment analysis of RELB target genes revealed significant involvement in NF-κB signaling, inflammatory response, and immune regulatory pathways (Figure 3F). This indicates that RELB exerts its function through these well-known pro-tumorigenic pathways.

To functionally validate the role of RELB, we knocked down RELB in neutrophils and co-cultured them with two tumor cell lines (A549 and H1299). This caused a reduction in the expression of NF-κB-associated and inflammatory genes IDO-1 and IL-6 within the tumor cells (Figure 3G). This suggests that RELB within neutrophils may modulate the tumor cell phenotype to be more inflammatory.

Furthermore, silencing RELB led to a significant reduction in tumor cell proliferation (Figure 3H), colony formation (Figure 3I), migration (Figure 3J), and invasion (Figure 3K) in both A549 and H1299 cell lines. These functional validations suggest that RELB activity in neutrophils may play an important role in enhancing the aggressive behaviors of tumor cells.

Collectively, these results suggest that RELB may function as a key transcription factor influencing the pro-metastatic behavior of the ELANE + Neu subpopulation, in part through activation of NF-κB and inflammatory pathways. This regulatory program is likely to contribute to lymph node metastasis in EGFR-wildtype LUAD.

### 3.4 High RELB signature indicates aggressive disease and poor outcomes in EGFR-wildtype LUAD

**FIGURE 4:**
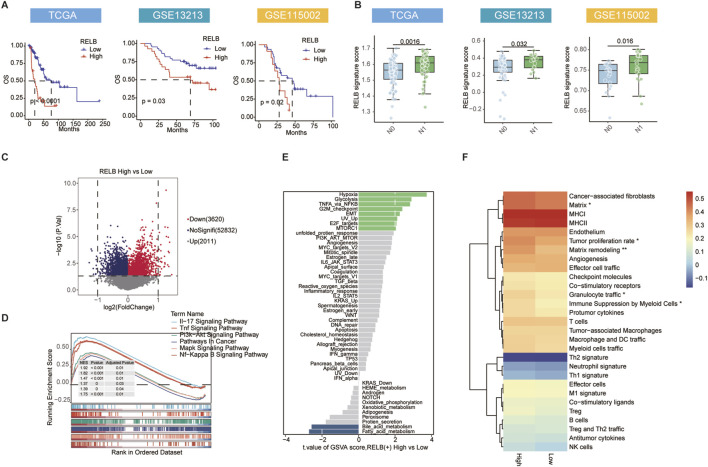
A high RELB signature score predicts poor prognosis and correlates with malignant features. **(A)** Kaplan-Meier curves for overall survival based on median RELB signature score in TCGA, GSE13213, and GSE115002 cohorts. **(B)** Comparison of RELB signature score across different N stages. **(C)** Volcano plot displaying DEGs between high and low RELB score groups in TCGA cohort. **(D)** GSEA showing significantly enriched KEGG pathways in high RELB score group. **(E)** GSVA for Hallmark pathways differentially enriched between high and low RELB score groups, ranked by t-value. **(F)** Heatmap depicting infiltration levels of 22 immune cell types in high versus low RELB score groups. Data are presented as mean ± SD. ns, not significant; ^*^ *P* < 0.05, ^**^ *P* < 0.01, ^***^ *P* < 0.001 by two-tailed t-test **(B)**. P values were determined by log-rank test **(A)**.

To evaluate the transcriptional output of the RELB pathway and its clinical relevance beyond the single-cell level, we constructed a RELB signature score based on its target gene set using the ssGSEA method and applied it to bulk transcriptomic data. In three independent EGFR-wildtype LUAD cohorts, a high RELB score consistently predicted shorter overall survival (Figure 4A). The score was significantly elevated in tumors with lymph node metastasis and advanced clinical stage (Figure 4B; Supplementary Figure S3), underscoring its close association with disease progression. We then stratified the TCGA cohort into high- and low-RELB score groups and performed differential expression analysis. The volcano plot illustrated considerable transcriptomic alterations in the high-score group as opposed to the low-score group, with 2011 genes upregulated and 3620 downregulated (Figure 4C), reflecting the broad regulatory impact of RELB on downstream gene expression.

The extensive influence emphasizes RELB as a primary regulator of tumor traits. GSEA of the upregulated genes showed significant activation across various pathways. The extensive influence emphasizes RELB as a primary regulator of tumor phenotype. Gene set enrichment analysis (GSEA) of the genes that were upregulated identified notable activation of various pathways, including the IL-17 signaling pathway, TNF signaling pathway, PI3K-AKT signaling pathway, pathways in Cancer, MAPK signaling pathway, and NF-κB signaling pathway (Figure 4D), indicating that RELB plays an important role in promoting inflammatory responses and cell survival pathways. The observed pro-tumorigenic functions align with this pathway activation profile. GSVA additionally confirmed the activation of hypoxia, EMT and other malignancy-related hallmark pathways in tumors with high scores (Figure 4E). Together, these analyses establish that high RELB activity is associated with a more aggressive tumor phenotype characterized by specific transcriptional reprogramming. Immune infiltration analysis showed that a high RELB score was correlated with features of an immunosuppressive microenvironment, characterized by enhanced myeloid-mediated immunosuppression, enrichment of pro-tumor cytokines, increased infiltration of cancer-associated fibroblasts, and reduced enrichment of immune-activation features such as anti-tumor cytokines and effector cells (Figure 4F). This suggests that the RELB signature captures an immunosuppressive TME state, potentially explaining its association with poor prognosis and metastasis.

In conclusion, this study demonstrates that the RELB signature score may serves as a prognostic biomarker and delineates a malignant tumor phenotype characterized by transcriptomic reprogramming, enhanced invasiveness, and immunosuppression in EGFR-wildtype LUAD.

### 3.5 The RELB signature predicts immunotherapy response and drug sensitivity

**FIGURE 5:**
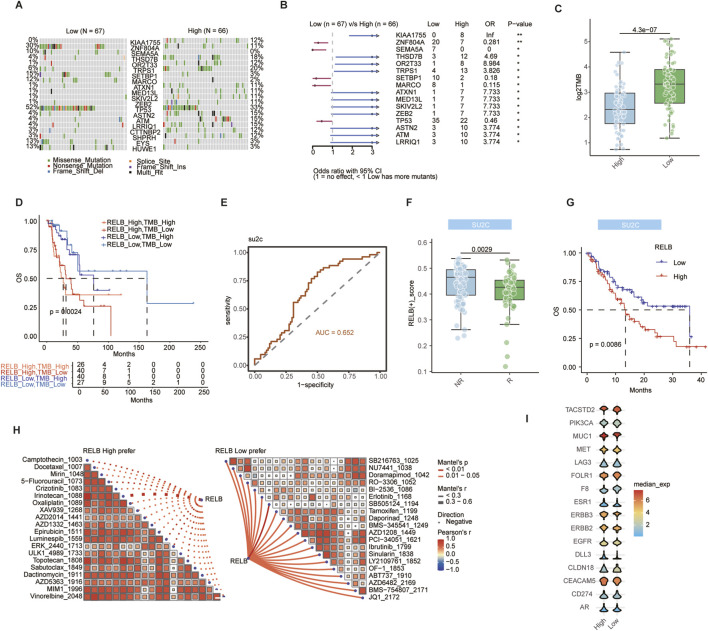
The RELB signature predicts immunotherapy response and drug sensitivity. **(A)** Oncoprint showing mutational landscape in high versus low RELB score groups within TCGA cohort. **(B)** Forest plot displaying difference in gene mutation frequency between high and low RELB score groups. **(C)** Box plot comparing Tumor Mutation Burden (TMB) between high and low RELB score groups in TCGA cohort. **(D)** Kaplan-Meier survival analysis of patients stratified by both RELB score and TMB. **(E)** ROC curve evaluating RELB score for predicting immunotherapy response in SU2C cohort, AUC = area under curve. **(F)** Box plot comparing RELB scores between responders and non-responders to immunotherapy in SU2C cohort. **(G)** Overall survival analysis based on optimal RELB score in SU2C immunotherapy cohort. **(H)** Correlation analysis between RELB score and drug IC50 values, showing top 20 negatively and positively correlated drugs. **(I)** Violin plots showing expression levels of ADC drug target genes in high versus low RELB score groups. Data are presented as mean ± SD. ns, not significant; ^*^ *P* < 0.05, ^**^ *P* < 0.01, ^***^ *P* < 0.001 by two-tailed wilcoxon test **(C,F)**. P values were determined by log-rank test **(D,G)**.

We next assessed the translational potential of the RELB signature in predicting therapy response. Distinct genetic modifications were identified in the EGFR-wildtype TCGA cohort’s mutational landscape analysis (Figures 5A,B), along with a significantly lower tumor mutational burden in the high RELB score group (Figure 5C). This inverse correlation indicates distinct biological differences between tumors with high and low RELB levels. By integrating the RELB score with TMB, patients were effectively categorized, revealing a subgroup with the worst prognosis (Figure 5D). This demonstrates the complementary prognostic value of RELB score and TMB. In the SU2C immunotherapy cohort (predominantly non-EGFR mutant), a low RELB score was associated with better therapeutic response, as confirmed by ROC analysis (Figures 5E,F), and longer overall survival (Figure 5G). This provides direct evidence that the RELB signature can predict inferior response to immune checkpoint inhibitors.

A drug sensitivity analysis was performed using the calcPhenotype function from the oncoPredict R package. It was found that there were several targeted anti-tumour agents that may be effective in patients with low RELB scores, such as NU7441, Doramapimod, AZD1208, etc. (Figure 5H). Patients with elevated RELB scores were sensitized to several chemotherapeutic agents (Docetaxel, 5-Fluorouracil, Irinotecan, Topotecan, etc.) and targeted anti-tumor agents (Crizotinib, Luminespib, AZD1332, MIM1, etc.) (Figure 5H). The differential pattern of drug sensitivity suggests potential treatment strategies that can be tailored according to the RELB activity status. Notably, several ADC drug targets were expressed at significantly higher levels in the high RELB score group (Figure 5I), suggesting a potential therapeutic vulnerability for this aggressive tumor subtype. This discovery suggests that ADCs could be a promising treatment option for tumors with high RELB expression that resist immunotherapy.

In conclusion, these findings indicate that the RELB signature may be a strong biomarker for forecasting prognosis, response to immunotherapy, and unique drug sensitivity patterns, emphasizing its potential in guiding personalized treatment strategies in EGFR-wildtype LUAD.

## 4 Discussion

This study systematically deciphered the cellular and molecular basis of lymph node metastasis in EGFR-wildtype lung adenocarcinoma by integrating multi-cohort single-cell transcriptomic data with bulk RNA-seq analyses. A pro-metastatic neutrophil subset, ELANE + Neu, was identified and found to be specifically enlarged in LNM samples. Further analysis revealed RELB as its primary transcriptional regulator. We subsequently developed a RELB-based gene signature, which stably predicts patient prognosis and immunotherapy respons. The findings enhance the insight into this challenging subtype and present potential biomarkers and new therapeutic strategies for patients with scarce treatment options.

In LNM samples of EGFR-wildtype LUAD, we identified a substantial increase in myeloid cells. This finding is consistent with the growing recognition of the pivotal function of myeloid cells within the tumour microenvironment. Numerous research efforts have demonstrated their significance in establishing an immunosuppressive setting and facilitating tumor development (Gu et al., 2025; Runtsch et al., 2025; Ramel et al., 2021). By precisely classifying patients based on EGFR status, our single-cell atlas underscores the particular involvement of myeloid cells, notably neutrophil subsets, in metastasis within this subtype. Further analysis through sub-clustering indicated a specific expansion of the ELANE + Neu neutrophil subset in LNM samples. This represents a substantial shift in the study of neutrophils, shifting from perceiving them as a single entity to understanding the specific functions of different neutrophil subpopulations in pre-metastatic niche development and immune regulation (Martin et al., 2025; Shi et al., 2025). Importantly, ELANE + Neu infiltration levels positively correlated with advanced N stage. There was a notable link between high infiltration and decreased overall survival in several independent cohorts. These findings highlight the clinical importance of ELANE + Neu as a major factor in metastasis for EGFR-wildtype LUAD.

By employing pySCENIC-based regulon analysis, we found that RELB serves as the primary transcription factor for the activation of ELANE + Neu cells. RELB plays a crucial role in the non-canonical NF-κB pathway, which is gaining recognition for its involvement in creating immunosuppressive conditions and fostering cancers linked to chronic inflammation (Ouyang et al., 2025; Navarro et al., 2025; Scherr et al., 2024). ELANE encodes neutrophil elastase, which can modulate downstream inflammatory signaling pathways, such as IL-8 and cell migration, through the TLR4-NF-κB pathway (Yang et al., 2021). The collected data pinpoint the activation of RELB in the pro-metastatic ELANE + Neu subpopulation, which provides fresh insight into the cell-specific functions of the NF-κB pathway in cancer (Lin et al., 2018).

In this study, the RELB signature score, derived from its target genes, served as an independent prognostic biomarker. Additionally, it identified a malignant tumor phenotype characterized by the activation of tumor-promoting pathways and the establishment of an immunosuppressive microenvironment. Previous studies have linked these detrimental traits to biological processes triggered by the activation of the NF-κB pathway (Scherr et al., 2024; Ok et al., 2015). The RELB score demonstrated a negative relationship with tumor mutational burden (TMB) and predicted poor immunotherapy outcomes. The progression of these tumors is typically driven by their own pro-metastatic signaling and an immune-suppressive setting. This finding corresponds to the traits of particular tumors that demonstrate restricted responsiveness to immune checkpoint inhibitors (Gu et al., 2025; Ma et al., 2025). Notably, our study found upregulated expression of several ADC drug targets in high RELB-score tumors. This point to a potential therapeutic direction for these aggressive tumors with limited treatment options, and antibody-drug conjugates targeting specific antigens are increasingly becoming an important clinical strategy (Bian et al., 2025; Ruan et al., 2025).

Despite offering comprehensive insights into the role of RELB-driven neutrophils in LNM of EGFR-wildtype LUAD, yet it is vital to acknowledge some limitations. *In vitro* experiments with neutrophil-tumor co-culture systems were primarily used for functional validation of RELB. Further *in vivo* studies are required to strengthen causal inference, such as those involving genetically engineered mice or patient-derived xenografts. The specific neutrophil effector mechanisms underlying these effects also need to be elucidated. Additionally, the absence of spatial transcriptomics, multiplex immunofluorescence or other spatially resolved multi-omics data prevents direct validation of the spatial colocalisation and physical interactions between ELANE + neutrophils and tumour cells. Future studies that integrate spatially resolved technologies will be essential for confirming cellular proximity and functional interplay. Furthermore, the clinical relevance of the RELB signature score, obtained from transcriptomic data, needs to be validated prospectively in various centers. Ultimately, although increased expression of multiple ADC targets was observed in tumors with high RELB expression, these findings are derived from transcriptomic analyses and lack protein-level or functional validation. The therapeutic relevance of these observations requires confirmation in appropriate preclinical models.

In summary, our research reveals a pro-metastatic neutrophil group, termed ELANE + Neu and regulated by RELB, as a primary contributor to lymph node metastasis in EGFR-wildtype LUAD. Additionally, we have formulated a RELB gene signature that serves as an effective biomarker, predicting patient prognosis, guiding immunotherapy responses, and identifying potential therapeutic vulnerabilities to antibody-drug conjugates. This research advances our knowledge of metastatic biology in this aggressive subtype and establishes a foundation for precision therapy.

## Data Availability

The original contributions presented in the study are included in the article/Supplementary Material, further inquiries can be directed to the corresponding authors.

## Supplementary material

SUPPLEMENTARY FIGURE S1 Single-cell enrichment analysis of LNM-associated gene signatures. **(A)** UMAP plots showing cells colored by dataset before (left) and after (right) batch effect correction using Harmony. **(B,D)** Feature plots visualising spatial distribution of enrichment scores for LNM-upregulated **(B)** and LNM-downregulated **(D)** gene sets on UMAP. **(C,E)** Violin plots comparing enrichment scores for LNM-upregulated **(C)** and LNM-downregulated **(E)** gene sets across different cell types.

SUPPLEMENTARY FIGURE S2 Comparison of ELANE + Neu infiltration scores across different stage in TCGA and GSE13213.

SUPPLEMENTARY FIGURE S3 Comparison of RELB signature score across different stages in TCGA and GSE13213.
