# Supplementary figures and images for: Single-cell atlas reveals a pro-metastatic RELB+ neutrophil-myeloid subset underlying lymph node metastasis in EGFR-wildtype LUAD
Source: Front Cell Dev Biol. 2026 Feb 17;14:1766211. doi: 10.3389/fcell.2026.1766211 (PMC12953111; doi:10.3389/fcell.2026.1766211)

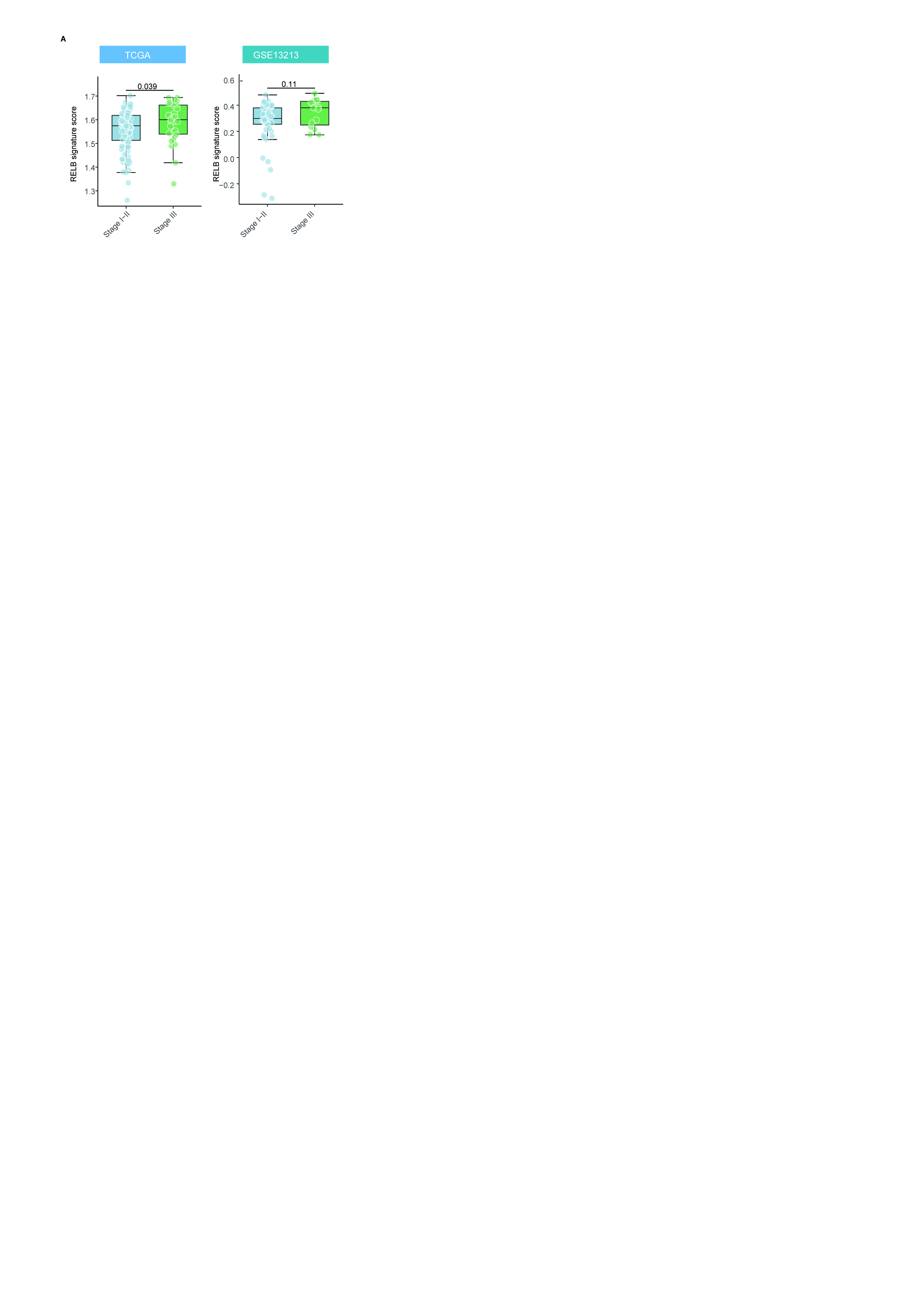

Supplement: Supplementary file 1 [file Image3.tif]

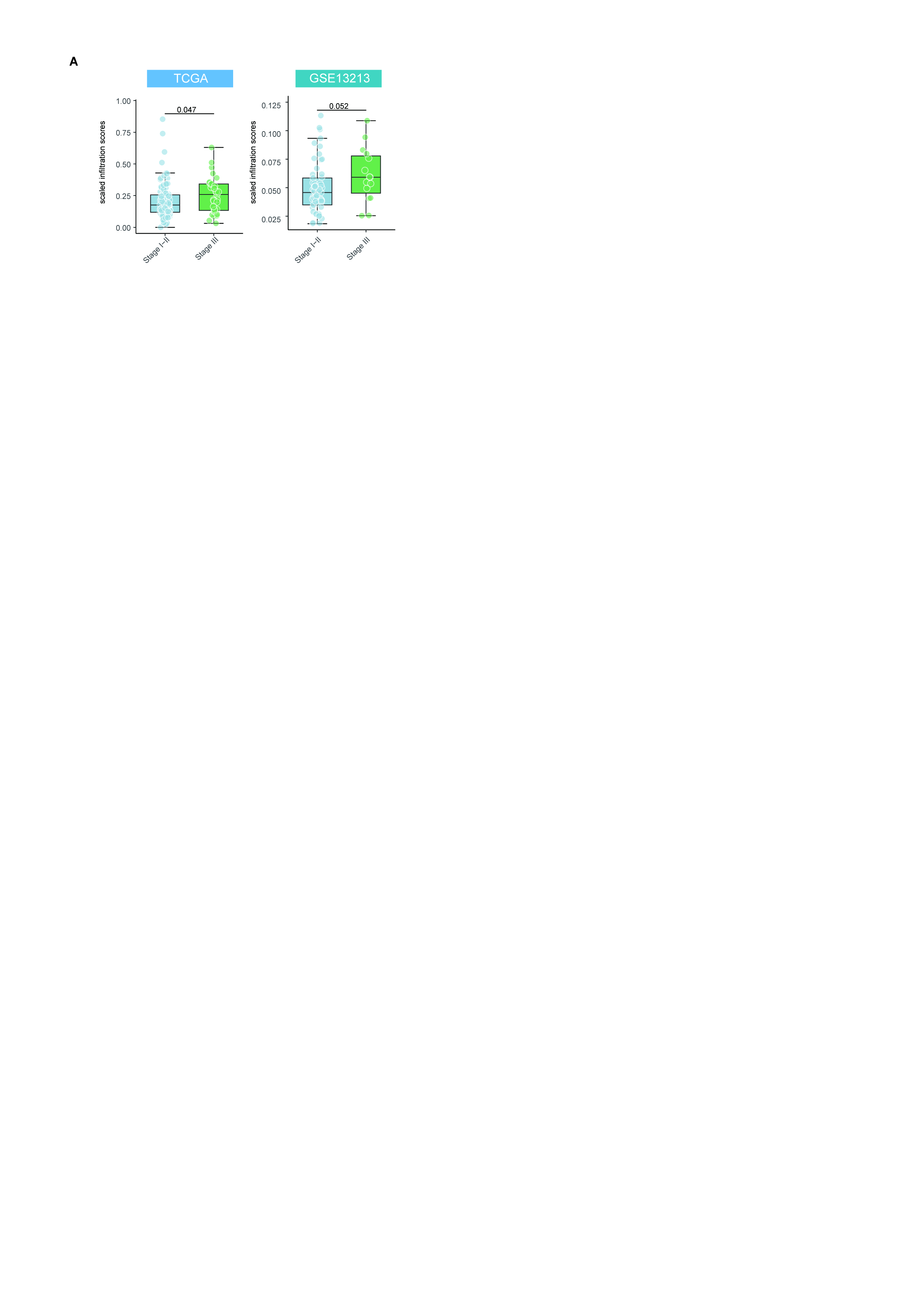

Supplement: Supplementary file 2 [file Image2.tif]

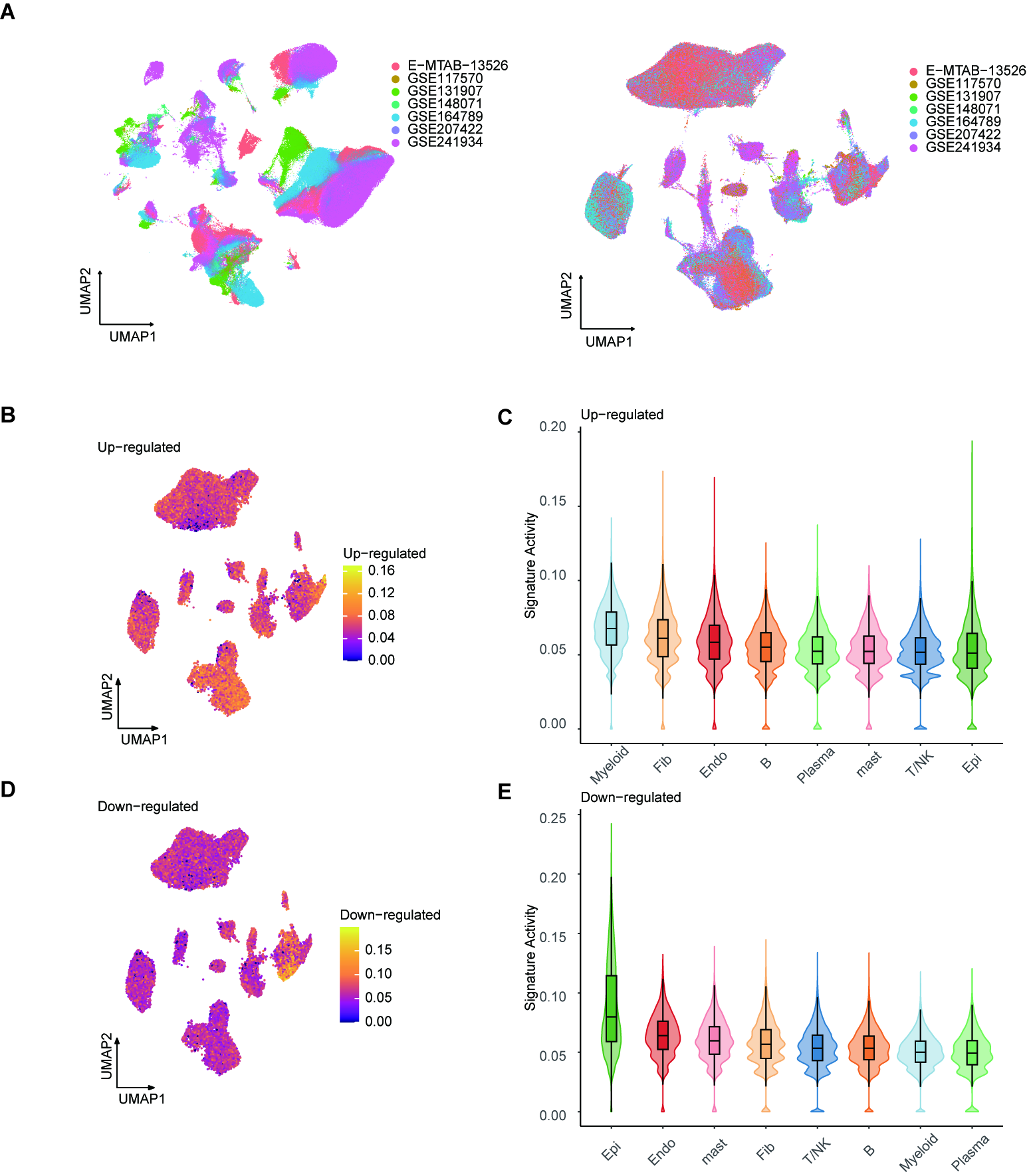

Supplement: Supplementary file 3 [file Image1.tif]
